# Profiling Long-Distance Urban Near-Surface Structures with Temporary Fiber-Optic Sensing in Jinan City, China

**DOI:** 10.3390/s26103118

**Published:** 2026-05-15

**Authors:** Lisong Chang, Weijun Wang, Kun Yan, Hengru Lv, Bosi Yang, Xun Wang, Feng Yang

**Affiliations:** 1 Key Laboratory of Earthquake Forecasting and Risk Assessment, Ministry of Emergency Management, Institute of Earthquake Forecasting, China Earthquake Administration, Beijing 100036, China; changlisong23@mails.ucas.ac.cn (L.C.);; 2 Hefei National Laboratory, Hefei 230088, China; 3 Institute of Geophysics, China Earthquake Administration, Beijing 100081, China

**Keywords:** Distributed Acoustic Sensing (DAS), urban subsurface structure, ambient noise interferometry, S-wave velocity imaging

## Abstract

**Highlights:**

**What are the main findings?**

- Using Distributed Acoustic Sensing (DAS) to efficiently record traffic vibrations on surface-laid optical cables, a 23 km seismic profile in Jinan was mapped and revealed a complex underground structure of alternating uplifts and grabens.
- The profile consists mostly of Class D soils with resonant frequencies spanning 1 Hz to 7 Hz due to uneven bedrock.

**What are the implications of the main findings?**

- Mobile DAS observation presents a new approach for cost-effective and rapid shallow imaging in noisy urban settings, without relying on existing fiber-optic cable resources.
- The substantial bedrock variations in Jinan are primarily attributed to early magma intrusions and have also been subjected to considerable faulting. This shallow morphology induces complex seismic site effects and has the potential to alter the pathways of the region’s abundant groundwater.

**Abstract:**

Fine-scale urban underground exploration is vital for geological safety and hydrogeological protection. In spring-rich cities like Jinan, shallow structures—such as sedimentary layers and fault systems—act as critical regulators of groundwater migration and spring formation. Yet, traditional seismic methods are often hindered by high costs and complexity. While Distributed Acoustic Sensing (DAS) offers a solution, its effectiveness is frequently limited by the poor coupling and coherent signal loss of existing cables in pipes. This study proposes an efficient alternative using mobile, unburied surface fiber-optic cables. Ten temporary DAS experiments were conducted along a 23 km line in Jinan, accompanied by nodal seismometers. Stable dispersion curves along the line can be extracted by subarray ambient noise interferometry with short-duration urban traffic noise DAS recording, and finally a high-resolution 2D S-wave velocity profile was mapped. The result shows that the profile has pronounced subsurface lateral heterogeneity, characterized by the alternation between two uplift zones and two grabens, which is highly consistent with H/V results from nodal seismometers. This confirms that mobile surface-cable DAS provides a rapid, reliable, and cost-effective imaging solution for characterizing complex urban subsurface structures, providing essential data for both geohazard assessment and the protection of groundwater transport pathways.

## 1. Introduction

The near-surface terrestrial environment constitutes a critical zone, hosting essential modern urban lifeline systems and vital public services, including subways, utility tunnels, and underground commercial complexes. Its structural stability directly governs a city’s operational safety and disaster prevention capabilities [1]. Shallow subsurface structures, particularly unconsolidated sedimentary strata and the underlying bedrock topography, serve as the physical basis for urban seismic wave amplification [2], soil liquefaction [3,4], and geological hazards such as subsurface cavities and ground subsidence. These shallow formations are also the primary determinants of the unique hydrogeological environment, regulating the migration and emergence of groundwater.

**Figure 1:**
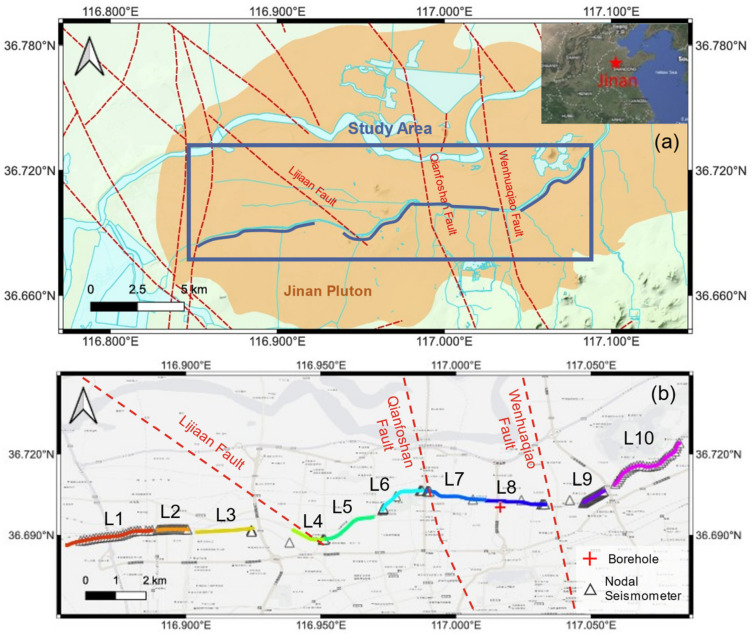
(**a**) Topographic map of Jinan. The blue lines and polygons represent rivers and lakes respectively. The blue box denotes the study area, while the red dashed lines indicate the faults, most of which are hidden. The orange area represents the Jinan Pluton. (**b**) Schematic map of the study area. L1–L10 represent the ten survey lines of the observations conducted in this study. Each colored thick line denotes a DAS session, while the triangle is the location of the nodal seismometer. The red pluses represent adjacent boreholes.

Jinan, the capital of Shandong Province in eastern China, is characterized by a unique hydrogeological framework situated at the transition zone between the southern mountainous terrain and the northern plains (Figure 1). The regional geological architecture can be conceptualized as an “impermeable plutonic barrier” intersecting a “karst aquifer system.” The southern region is dominated by Cambrian–Ordovician limestone with highly developed fissures and karst conduits, serving as a high-permeability recharge aquifer. Conversely, the north-central urban area is underlain by the Jinan Pluton—a massive, concealed igneous body composed primarily of impermeable gabbro and diorite. As groundwater migrates northward, driven by topography, this magmatic massif acts as a formidable subsurface barrier, truncating horizontal flow and forcing the deep-seated karst water to upwell through thin Quaternary windows, thereby forming the city’s renowned spring clusters [5].

The fine-scale morphology of this bedrock is governed by a complex “horst-and-graben” structural framework resulting from early magmatic intrusions and subsequent tectonic modification. The NNW-trending faults, specifically the Qianfoshan Fault and the Wenhuaqiao Fault, have segmented the Jinan Pluton and its overlying Quaternary deposits into a series of alternating uplifts and depressions. In this study, our DAS imaging reveals a distinct “two-uplift, two-graben” configuration. These structural undulations are not merely topographic variations but serve as the primary regulators of groundwater dynamics: the high-velocity uplift zones act as impermeable subsurface dams, while the velocity discontinuities and fractured zones associated with faulting provide potential preferential pathways or boundaries for spring water migration. Therefore, high-resolution mapping of these S-wave velocity contrasts is imperative, as they delineate the precise geometry of the magmatic “barrier” and the spatial distribution of the “conduits,” providing a critical physical basis for both groundwater conservation and urban geohazard assessment.

Constructing a high-resolution shallow structure will also contribute to the city’s earthquake safety. Jinan has a history of earthquakes up to magnitude 5, and there are several faults in the city’s vicinity capable of generating earthquakes up to magnitude 7. Furthermore, Jinan has a low sedimentary thickness and is rich in surface water. The urban seismic site effects may be amplified not only by high-frequency resonance but also by the possibility of soil liquefaction. These two factors have a relatively large impact on buildings, leading to a high risk of earthquake damage. A recent case is the M5.5 earthquake that struck Pingyuan County on 6 August 2023. Although it was about 100 km from Jinan, it still caused significant tremors in the city and resulted in severe panic among the people.

However, implementing fine-scale geological exploration within highly urbanized environments encounters significant challenges. Traditional active seismic methods are constrained by densely packed infrastructure and transportation networks, compounded by pervasive anthropogenic noise emanating from busy traffic and industrial operations. Ambient noise imaging offers a solution by utilizing this anthropogenic noise, emerging as a primary tool for urban subsurface investigation [1,6]. Achieving high-resolution ambient noise imaging necessitates dense acquisition arrays, often on a meter scale. Such dense arrays typically employ nodal seismometers, characterized by their portability, cost-effectiveness, and capacity for extensive spatial coverage. However, deploying nearly a thousand nodal seismometers simultaneously for underground imaging over a distance of 1–2 km represents an exceptionally labor-intensive and time-consuming undertaking.

In recent years, advancements in Distributed Acoustic Sensing (DAS) technology have provided a new technical pathway for urban underground space exploration [7]. DAS transforms fiber-optic cables into thousands of distributed sensors, enabling meter-scale continuous sampling and long-term vibration monitoring over distances up to 100 km at a relatively low cost [8,9]. Currently, this technology is widely applied in various fields, including oil and gas resource monitoring [10,11], traffic vibration identification [12,13,14], natural earthquake monitoring [15], and near-surface imaging [16,17,18,19,20,21,22,23]. Its high spatial resolution and continuous monitoring characteristics make it particularly suitable for fine-scale exploration under complex urban geological conditions and high-interference environments.

One advantage of DAS applications is their capacity to utilize existing fiber-optic communication resources in urban areas. This significantly reduces equipment deployment and maintenance costs while enabling long-distance synchronous data acquisition [17,24,25,26,27,28]. Currently, most shallow underground imaging applications using DAS rely on dark optical fibers in available communication cables [29,30,31,32,33]. Although this approach is cost-effective, there are still some concerns regarding the rapid utilization of the vast amount of optical fiber resources in cities. The ground coupling of fiber-optic cables is a major issue, as almost all cables are loosely installed in underground pipes. The coupling may be at its worst when the pipe is surrounded by other pipes in a hole. Poor ground coupling and continuous variation along the optical fiber make it challenging to extract high-quality surface wave signals. Another problem is the difficulty in quickly and accurately determining the route location of the optical cable. Especially when the optical cable duct is buried deep underground and the optical cable coupling is poor, using the tap-test to locate the cable becomes a very time-consuming task. Therefore, the challenge of balancing construction efficiency with detection quality remains a critical bottleneck restricting the large-scale promotion of DAS technology.

Consequently, to achieve a more profound comprehension of the shallow structure in Jinan, this study undertakes high-resolution imaging while addressing the common bottleneck of poor ground coupling in pre-existing urban fiber networks. We implemented a mobile observation strategy, involving the rapid deployment of temporary fiber-optic cables directly onto surface walkways. This approach ensures direct mechanical coupling and maximizes signal sensitivity to urban traffic vibrations, enabling the extraction of high-quality surface waves with a limited recording duration.

Crucially, our workflow does not rely on a single data source; instead, we establish a multi-parameter validation framework. The DAS-derived S-wave velocity structures are systematically cross-validated against independent microtremor H/V spectral ratios from nodal seismometers and stratigraphic data from deep boreholes. This integrated approach ensures that the identified velocity undulations represent real geological features rather than inversion artifacts. The resulting 2D profile provides a high-resolution “tectonic skeleton” of the urban subsurface, offering essential data for evaluating seismic site effects and delineating groundwater migration pathways in this spring-rich environment.

## 2. Data and Methods

### 2.1. Data Acquisition

**Figure 2:**
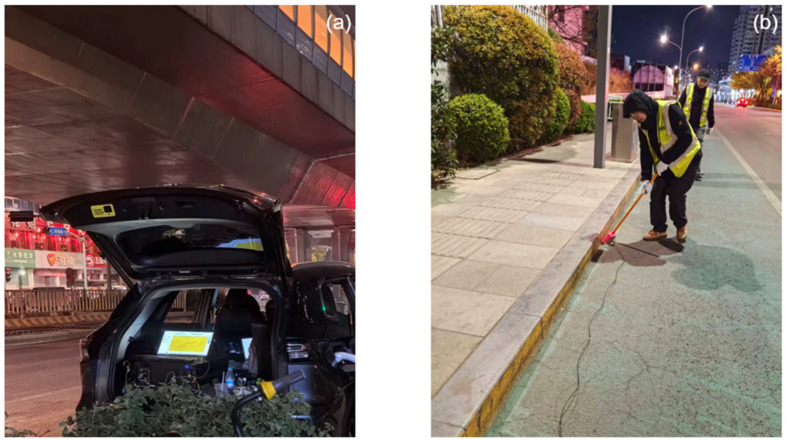
(**a**) Running DAS interrogator in car; (**b**) fiber-optic cable field deployment.

The experimental survey line for this study, comprising ten DAS observation segments, is situated along the riverside walkway of the Xiaoqinghe River in Jinan, extending from Nanbei No. 3 Road in the west to Caiyuan Bridge in the east (Figure 1). For each segment, a 1–3 km fiber-optic cable was deployed directly onto the walkway surface without burial or anchoring. This approach facilitated rapid deployment and ensured direct mechanical coupling with the rigid asphalt/concrete pavement, which effectively avoids the “pipe-in-pipe” decoupling effect common in buried telecommunication ducts. Cable positions were determined using differential GNSS positioning along the route. One end of each cable was interfaced with the DAS interrogator, which was housed within and powered by an electric vehicle (Figure 2a). Two interrogator models, the Silixa iDAS (v2.4) (Silixa Ltd., Elstree, UK) and the ZD-DAS (Zhidi Perception (Hefei) Technology Co., Ltd., Hefei, China), were utilized for data acquisition. The Silixa iDAS was configured with a 50 ns pulse width and a 10 m gauge length, while the ZD-DAS utilized a 30 ns pulse width and a 6 m gauge length, which can achieve a finer spatial resolution but typically comes with lower sensing sensitivity. Both systems set the spatial sampling interval to 2.04 m and the laser pulse rate to 8000 Hz. The data was further downsampled to 1000 Hz and saved to disks. Additionally, three-component nodal seismometers were deployed along the fiber-optic segments at irregular intervals; deployment was dense along segments L1, L2, L9, and L10, yet sparse for the remaining segments due to localized traffic control and site accessibility; nevertheless, the 1–2 m channel spacing of DAS maintains a spatial resolution far superior to that of traditional nodal arrays. Observation for each segment lasted at least three hours, from which a 1.5-h high-quality segment was selected for noise interferometry to extract stable dispersion curves, and the entire survey line consisted of a total of 19 km of fiber-optic cable.

The sensing element adopted in this study was an armored tactical fiber-optic cable with an outer diameter of 4 mm. The cable is approximately 20 kg/km and has excellent longitudinal mechanical performance. As shown in Figure 2b, the cable was deployed along the road surface without trenching. The self-weight of the cable, combined with the micro-roughness of the asphalt or concrete pavement, would provide sufficient mechanical friction for effective strain transfer.

The coupling quality between the fiber and the pavement can be directly evaluated via tap-test or moving vehicle signals, which are shown by their consistency in amplitude and phase along the cable. The tap-test is only used for the surrounding optical cables at specific points, but the whole optical cable can be checked by the vehicle track. Coupling usually affects amplitude, but phase is mainly used in seismic noise imaging. Therefore, in the strong urban traffic noise environment, the loss of amplitude caused by coupling has little impact on noise imaging. Moreover, the seismic array processing technology used in this paper can also suppress some poorly coupled signals in the optical fiber. Hence, the ambient noise cross-correlation functions and the dispersion curves derived from DAS data are other criteria to evaluate the coupling. According to the results in Section 3, the surface-laid implementation provided reliable coupling for frequency ranges of at least 1–20 Hz.

### 2.2. Data Processing Methods

#### 2.2.1. Nodal Data Processing

The deployment of nodal seismometers was insufficiently dense to extract high-frequency surface waves; consequently, the microtremor horizontal-to-vertical spectral ratio (HVSR) technique was applied to the three-component data. The microtremor HVSR method is extensively employed in seismic microzonation studies [34,35,36,37,38,39]. The resonance frequency and the thickness of the unconsolidated sedimentary layer derived from HVSR analysis provide essential verification for subsequent subsurface velocity inversion. Key information revealed by the H/V spectral ratio curves, such as the predominant frequency and amplification factor, not only characterizes the dynamic properties of the site but also furnishes a valuable a priori constraint for interpreting dispersion curves. Integrating HVSR results with velocity structure models effectively optimizes exploration workflows for near-surface structures and enhances inversion accuracy [40].

The calculation formula for the H/V spectral ratio is as follows:

(1) $${\left(\frac{H}{V}\right)}_{s}\left(f\right)=\frac{H\left(f\right)}{Z\left(f\right)}=\;\sqrt{\left[\frac{\left(P_{NS}^{2}\left(f\right)+\;P_{EW}^{2}\left(f\right)\right)}{\left(2P_{UD}^{2}\left(f\right)\right)}\right]}$$

where *P_NS_*(*f*), *P_EW_*(*f*), and P_UD_(*f*) denote the Fourier amplitude spectra recorded by the seismometer in the north–south, east–west, and vertical directions, respectively. When a significant wave impedance contrast exists in the shallow subsurface, the H/V spectral ratio curve exhibits one or more peaks, which can be utilized to estimate the depth of the interface.

It is widely accepted in existing research that the peak frequency of the H/V spectral ratio curve approximates the predominant frequency of the soil layer—that is, the resonance frequency of the site’s shear waves. The quantitative relationship between the resonance frequency of the soil layer, the average shear-wave velocity, and the thickness of the sedimentary layer can be expressed by Equation (2). Consequently, given the shear-wave velocity background of the study area, the thickness of the sedimentary layer can be inverted using Equation (2) [41,42]:

(2) $$f_{p}\approx f_{s}=V_{\mathrm{sa}}/(4h)$$

where V_sa_ is the average S-wave velocity of the soil layer and *h* is the layer thickness. Generally, the average velocity of soil layers exhibits an exponential increase with depth; thus, the relationship between the resonance frequency (*f_s_*) and the thickness of the unconsolidated sediments (*h*) can be expressed as follows [41,42]:

(3) $$h\;=\;a{f_{s}}^{b}$$

where the parameters *a* and *b* are typically obtained by fitting a dataset of unconsolidated sediment thicknesses and resonance frequencies. Furthermore, based on Equations (2) and (3), the relationship between the average soil layer velocity and thickness can also be established as follows [43]:

(4) $$V_{\mathrm{sa}}\;=\;e^{(\ln(h)+\mathrm{bln}(4h)-\ln(a))/b}$$

Therefore, when a reliable subsurface velocity structure is available, *a* and *b* can also be fitted according to Equation (4). The data processing workflow was primarily implemented using the Geopsy software (v3.5.2) [44], with the specific steps as follows.

**Figure 3:**
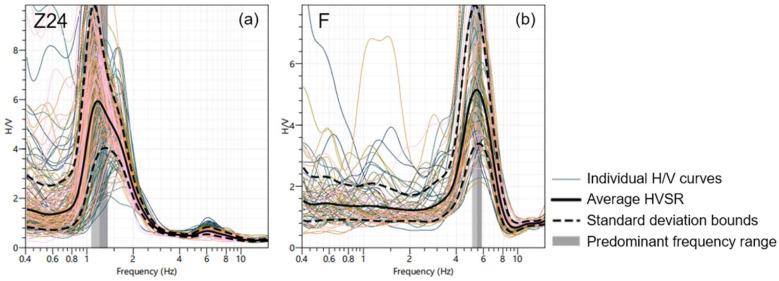
H/V spectral ratio curves of two nodal stations. The solid black line represents the average H/V curve across all data windows, and the two dashed black lines indicate the standard deviation range (confidence interval). The thin, colorful lines in the background are the original H/V curves extracted from individual time windows. The vertical gray-shaded area marks the identified predominant frequency (peak frequency) range of the station. (**a**) H/V spectral ratio of station Z24 on line L2; (**b**) H/V spectral ratio of station F on line L5.

First, transient signals were excluded using the Short-Term Average to Long-Term Average (STA/LTA) ratio. Steady-state noise windows with a length of 120 s were selected for H/V curve calculation using a sliding window approach with a 50% temporal overlap. The LTA (Long-Term Average) window was set to 100 s, the STA (Short-Term Average) window was set to 1.0 s, and the STA/LTA ratio threshold was set between a minimum of 0.2 and a maximum of 2.5. Subsequently, the H/V curves from each window were smoothed using the Konno & Ohmachi (1998) method [45]. The smoothing constant (b-value) was set to 40 to ensure uniform smoothing resolution across a logarithmic frequency axis and to effectively suppress high-frequency random noise. Finally, all time windows were averaged to obtain the mean H/V curve (Figure 3).

#### 2.2.2. DAS Data Processing

**Figure 4:**
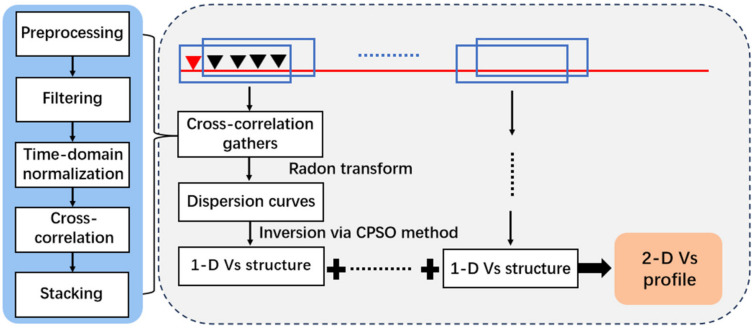
The workflow for DAS data processing and S-wave velocity structure inversion. The red line represents the fiber-optic cable, and the red and black triangles denote the virtual source and receiver channels, respectively.

Figure 4 illustrates the workflow for the DAS data processing in this study, encompassing four primary stages: preprocessing, cross-correlation calculation, dispersion curve extraction, and S-wave velocity inversion.

The preprocessing and cross-correlation complied with a standard ambient noise procedure [46]. Initially, the raw data were downsampled from 1000 Hz to 125 Hz, and anomalous channels were removed. Subsequently, frequency-domain spectral whitening and 1 to 20 Hz band-pass filtering were carried out. To eliminate clutter and interference signals from specific directions, a frequency–wavenumber (f–k) domain filter with a velocity range of 150–400 m/s was additionally applied.

The preprocessed data were segmented using a sliding time window of 60 s with a 50% overlap, and cross-correlation functions for all channels were calculated and normalized within each window. To enhance the signal-to-noise ratio (SNR) and generate high-quality virtual shot gathers, a multi-stage stacking strategy was implemented: first, cross-correlation functions were grouped by offset bins and linearly stacked [47]. Building upon this, a power-of-two (power = 2) phase-weighted stacking (PWS) algorithm was introduced to effectively suppress random noise with phase inconsistencies by leveraging the instantaneous phase coherence of the signals. Finally, the positive and negative time lags were symmetrically averaged to obtain the final noise cross-correlation functions (NCFs). In urban environments, 1–2 h of DAS records are sufficient to effectively extract dispersion curves [48]; therefore, 1.5 h of observation data were stacked in this study.

The NCFs can be further processed with methods like Multichannel Analysis of Surface Waves (MASW) to extract dispersion curves. Since DAS technology primarily senses axial strain (or strain rate), the signals recorded by horizontally deployed fiber optics essentially reflect the radial component of Rayleigh waves [49]. Based on the diffuse field approximation theory, for a horizontally layered isotropic medium, the relationship between the ambient noise cross-correlation function (NCF) of two stations (separated by distance r) and the imaginary part of the Green’s function is expressed as follows:

(5) $$C_{RR}\left(r,\omega \right)=A\cdot Im\left[\tilde{G_{RR}}\left(r,z=0;\omega \right)\right]\;\;$$

where CRR is the radial-component cross-correlation function in the frequency domain, $\tilde{G_{RR}}$ is the corresponding radial component of the Green’s function, and A is a constant coefficient.

To extract the dispersion energy, we applied the Radon transform to the processed NCF gathers [49]:

(6) $$m\left(p,\omega \right)=\int C_{RR}\left(r,\omega \right)\times e^{\left(i\omega pr\right)}dr$$

where m(*p*,*ω*) represents the Radon transform result in the frequency–slowness (ω−p) domain, and e^(*iωpr*)^ is the phase-shift operator within the Fourier transform, which implements the linear time shift *t* = *pr* in the frequency domain. Subsequently, cubic weighting was applied to the generated dispersion spectrum to enhance energy aggregation, resulting in high-resolution dispersion images. The dispersion curves were then precisely picked from the high-energy ridges of the dispersion energy spectrum using a semi-automatic method.

The one-dimensional S-wave velocity structure beneath each subarray was inverted based on the extracted dispersion curve. In this study, the Cooperative Particle Swarm Optimization (CPSO) algorithm was employed [50,51]. To strike a reasonable balance between computational efficiency and the quality of dispersion imaging, each subarray is 200 m in length and slides with a 60 m step to cover the whole cable (Figure 4). The processing was repeated for 10 DAS segments. Finally, a two-dimensional S-wave velocity profile along the fiber-optic lines was constructed by spatially concatenating the 1D inversion results and applying Kriging interpolation [52].

## 3. Results

### 3.1. DAS Data Characteristics

**Figure 5:**
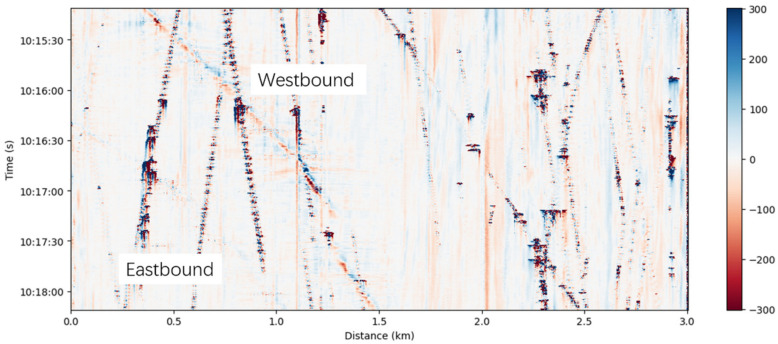
The waterfall plot for 3-min DAS recording in L10 session.

Figure 5 displays a 3-min segment of dense waveform records acquired by the DAS system. Distinct and dense trajectories of vehicles traveling both eastward and westward are clearly observable, identifying traffic as the predominant vibration source in this region. The clusters of waveforms visible within these vehicle trajectories likely represent signals generated by vehicles traversing uneven road surfaces or road irregularities. Furthermore, the variations in signal intensity along the survey line are largely attributed to the non-uniform ground coupling of the fiber-optic cable. However, overall, these amplitude fluctuations exert a relatively minor impact on the vibration phases and do not adversely affect the calculation of the noise cross-correlation functions (NCFs).

**Figure 6:**
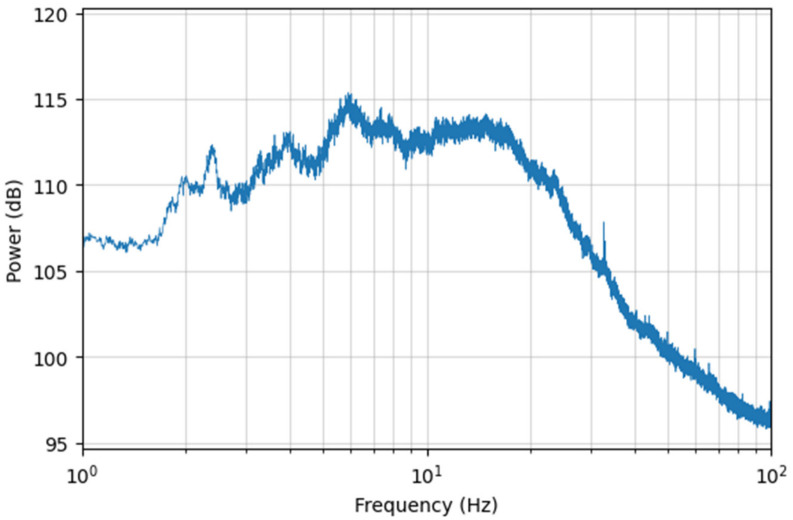
Average noise power spectra of all channels.

The average power spectra of the 3-min DAS records at various locations along the fiber-optic cable are shown in Figure 6. The noise energy is primarily distributed within the 1–40 Hz range, with peaks concentrated between 5 and 17 Hz, consistent with the characteristics of traffic-induced noise [32].

### 3.2. H/V Results

**Figure 7:**
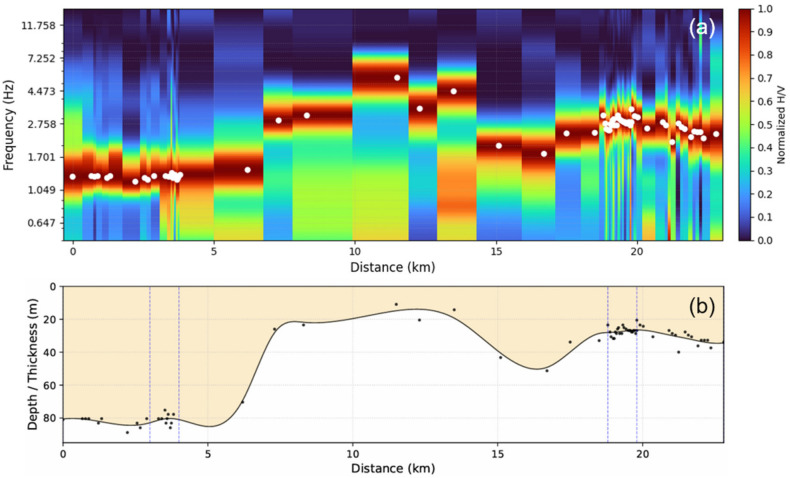
(**a**) Normalized microtremor H/V spectra along the fiber-optic survey line from west to east. (**b**) The loose sediment thickness profile calculated based on the empirical formula, where the nodal seismometers are non-uniformly distributed.

Seventy horizontal-to-vertical spectral ratio (HVSR) curves were acquired along the Xiaoqinghe River. These curves are normalized and plotted from west to east, as depicted in Figure 7a. All the HVSR curves exhibit a prominent peak, which shows significant spatial fluctuations along the river. Subsequently, the sedimentary thickness (*h*) was computed from these peak frequencies (*f_s_*) using the empirical formula provided below [23]:

(7) $$h=112.1f_{s}^{-1.377}$$

And the entire sedimentary layer was interpolated from these converted thicknesses using the univariate cubic spline smoothing method with a smoothing factor of s = 150, as shown in Figure 7b.

As illustrated in Figure 7, the predominant frequencies within the western section (0–4 km) are relatively subdued, primarily concentrated within the 1.0–2.0 Hz band. In conjunction with a priori geological knowledge constraining the thickness of deep sedimentary layers in this region, this frequency distribution indicates a substantial sedimentary cover, with depths predominantly fluctuating between 70 and 90 m. Upon transitioning to the middle section (7–15 km), the predominant frequencies exhibit a marked increase, exceeding 4.0–7.0 Hz. This characteristic aligns remarkably with the significant bedrock uplift trend observed through surface investigations. By applying boundary constraints indicative of shallowly buried bedrock, the fitted curve reflects a pronounced reduction in sedimentary thickness, confined within 20 m, thereby presenting a distinct bedrock uplift morphology. Within the eastern section (beyond 18 km), spatial cross-constraints derived from high-density measurement points reveal more refined lateral heterogeneity. Consequently, the HVSR distribution intuitively delineates the undulating morphology of the concealed basement, characterized by a “west-low, east-high, and central-convex” relief.

### 3.3. DAS Ambient Noise Imaging

#### 3.3.1. DAS Ambient Noise Cross-Correlation and Dispersion Curves

**Figure 8:**
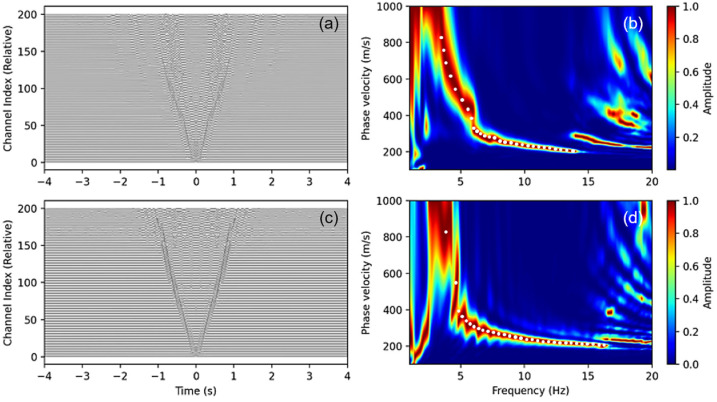
The cross-correlation functions (left panel) and dispersion spectra (right panel) derived from Distributed Acoustic Sensing (DAS) data. Graphs (**a**,**b**) present the NCFs for channels spanning 490–690 m on line L3, while (**c**,**d**) present the corresponding results for channels 430–630 m on line L6. The white dots depict the picked dispersion curve.

When optical fiber is deployed directly adjacent to roadways, the substantial traffic noise captured by Distributed Acoustic Sensing (DAS) proves advantageous for surface wave signal extraction. High signal-to-noise ratio ambient noise cross-correlation functions (NCFs) can be identified following brief stacking periods, as illustrated in Figure 8a,c. These figures depict the NCFs for the 490–690 m subarray of line L3 and the 430–630 m subarray of line L6, respectively. Distinct Rayleigh wave signals are discernible in both positive and negative time lags, exhibiting phase velocities spanning 200 m/s to 1000 m/s. Figure 8b,d display the corresponding dispersion spectra, wherein prominent fundamental-mode, first-higher-mode, and even higher-mode dispersion energy are identifiable. The fundamental mode operates within a frequency range of approximately 2–15 Hz, with phase velocities distributed between 0.2 and 1.0 km per second.

**Figure 9:**
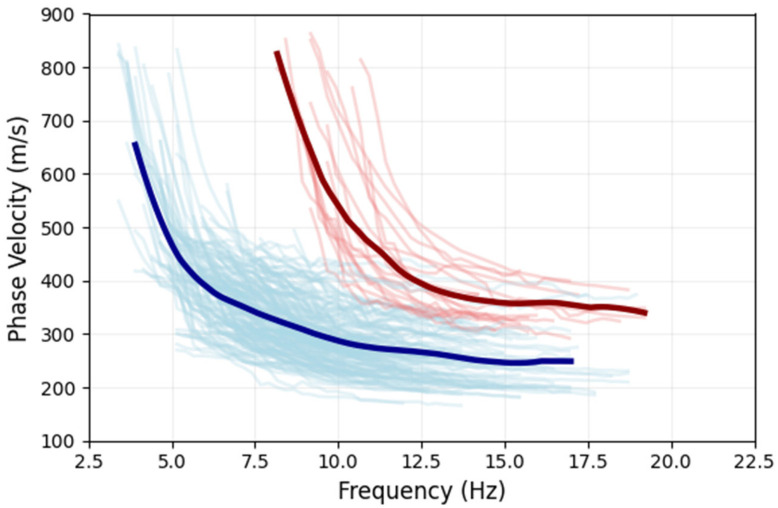
The fundamental-mode Rayleigh wave dispersion curves were extracted from all DAS subarrays. Light red curves correspond to those obtained from the L5 DAS data, whereas light blue curves designate the remaining DAS datasets. Bold lines delineate the mean values.

Ultimately, 286 dispersion curves were extracted using a 200 m long subarray window and a 60 m sliding step from ten DAS survey segments. Figure 9 illustrates all Rayleigh wave phase velocity dispersion curves extracted, alongside their average trends. These curves cluster into two distinct categories, exhibiting significantly disparate phase velocity distributions. The curves characterized by higher phase velocities primarily originate from line L5. This segment displays a narrower effective frequency band, with a minimum extractable frequency of approximately 7.5 Hz; its phase velocities within the high-frequency range (>15 Hz) are predominantly concentrated between 300 and 400 m/s. In contrast, other dispersion curves demonstrate broader low-frequency coverage. Their starting frequencies extend down to approximately 3.0 Hz, while their high-frequency phase velocities are distributed within a lower range of 200–300 m/s. This division of the dispersion curves suggests abrupt lateral variations in shallow subsurface structure adjacent to line L5.

#### 3.3.2. Velocity Structure Inversion

According to Figure 9, the maximum Rayleigh wave wavelengths derived from distinct DAS subarrays range from approximately 60 m to 200 m. Given the physical criterion that Rayleigh wave inversion depth corresponds to approximately 0.5–0.67 times the maximum wavelength, the inversion depth must be dynamically adjusted within a range of 40 to 100 m, corresponding to their respective available dispersion ranges. For example, within the L5 segment, likely related to shallow bedrock, the dispersions at low frequencies are difficult to identify, restricting their capacity to image deeper structures. To provide a uniform representation of deep structural features across the entire line, a Vs velocity profile extending to a depth of 100 m was ultimately obtained through spatial interpolation.

**Figure 10:**
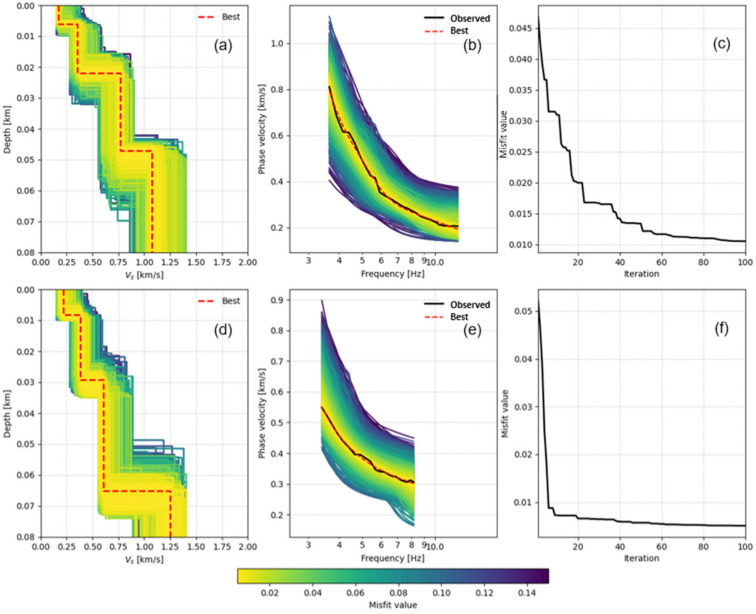
The dispersion curve inversions for subarrays L5 (**a**–**c**) and L8 (**d**–**f**) are presented. The L5 subarray extends from 490 m to 690 m, while the L8 subarray occupies the interval between 1390 m and 1590 m. Graphs (**a**,**d**) present the inverted S-wave velocity structure models, with the optimal best-fitting model delineated by a red dashed line. Graphs (**b**,**e**) depict the phase-velocity dispersion-curve fitting: the observed dispersion curve, extracted from measured data, is represented by a solid black line; the theoretical dispersion curve corresponding to the optimal model is denoted by a red dashed line; and dispersion curves derived from all sampled models appear as colored lines. Graphs (**c**,**f**) illustrate the convergence curves of the objective function (misfit) during the inversion process.

Two inversion cases for subarrays in L5 and L8 are presented in Figure 10. Initial S-wave velocity models composed of four horizontal layers are constructed from the dispersion curves. A Competitive Particle Swarm Optimization (CPSO) algorithm with global optimization capability was utilized for the inversion. The parameters were set such that the population size was 30 and the maximum number of iterations was 100. The misfit values quickly drop within the first 20 iterations and are nearly stable after 80 iterations (Figure 10c,f). The models during the iteration and their corresponding dispersion curves are plotted in the left and middle columns in Figure 10.

The inverted S-wave velocity structures exhibiting minimal misfit error reveal a pronounced transition from low velocities (<200 m/s) to high velocities (>1000 m/s) with increasing depth (Figure 10a,d). Their corresponding Rayleigh wave dispersion curves closely approximate the observed dispersion curves (Figure 10c,e). Laterally, the S-wave velocity structure for L5 consistently exceeds that of L8 across different depths. Furthermore, L5 demonstrates a significant velocity discontinuity at approximately 22 m depth, whereas L8 exhibits a similar discontinuity near 65-m depth. This velocity discontinuity likely corresponds to the sediment base, indicating that the deposition at L5 is relatively shallow. These findings exhibit characteristics consistent with the HVSR results.

**Figure 11:**
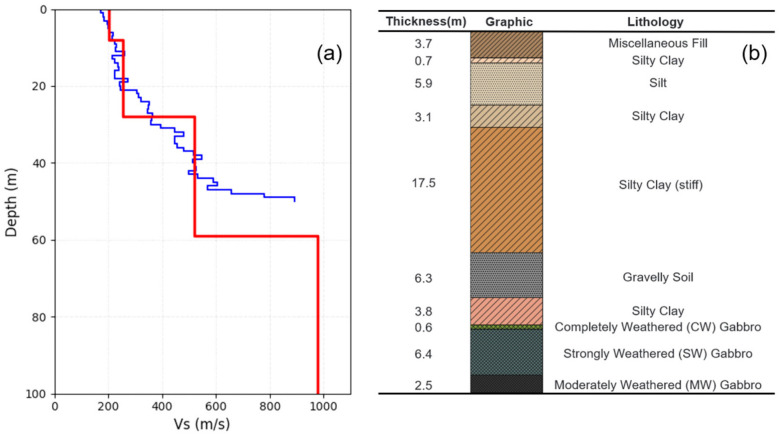
Velocities and strata in borehole adjacent to L8. (**a**) Borehole S-wave velocity (Vs) profile (blue line) and inverted S-wave velocity profile in nearby DAS subarray (red line). (**b**) Stratigraphic map of the borehole.

Figure 11 presents the velocity profile and stratigraphic map for a borehole situated approximately 253 m adjacent to L8 (Figure 1). The borehole data reveal that the strata comprise sediments, predominantly clay, within the initial 41 m, succeeded by approximately 7 m of completely or intensely weathered gabbro, and ultimately unweathered gabbro. Consequently, the pronounced velocity jump observed near the 50-m depth likely corresponds to the interface between the weathered and unweathered gabbro layers. The inverted S-wave velocities derived from the subarray proximal to the borehole are also illustrated in Figure 11, demonstrating a strong correlation with the borehole velocity profile.

#### 3.3.3. S-Wave Velocity Profile Along the Xiaoqinghe River

**Figure 12:**
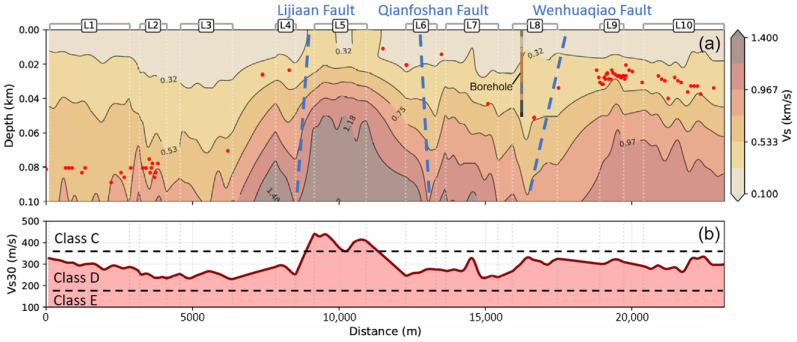
(**a**) Two-dimensional shear-wave velocity (Vs) profile from west to east. The red dots represent the sedimentary thicknesses calculated from the H/V peak frequencies. The blue dashed lines indicate the predicted fault locations. (**b**) The time-averaged shear-wave velocities in the top 30 m of the subsoil (Vs30) along the profile. The dashed horizontal lines draw the soil types according to the NEHRP scheme.

By interpolating the 1D velocity structures inverted from each subarray, a 2D S-wave velocity profile down to a depth of approximately 100 m beneath the survey line was obtained (Figure 12a). Overall, the S-wave velocity exhibits an increasing trend with depth and demonstrates significant lateral heterogeneity. The fluctuations in sedimentary thicknesses obtained from H/V and the S-wave velocity variations demonstrate a high degree of spatial correlation.

Within the shallow section at depths shallower than 20 m, medium velocities are generally relatively low (<0.4 km/s), with lateral horizons exhibiting relative continuity and gentleness. Beyond 40 m depth, however, intense lateral fluctuations in wave velocity become apparent. Notably, a pronounced bulge morphology is observed between distances of 8000 m and 12,000 m (corresponding to survey segments L4 and L6). Here, high-velocity contours (e.g., 0.75 km/s) rise rapidly from approximately 80 m depth on either side to about 40 m depth, forming a distinct high-velocity ridge. A similar, though less pronounced, bulge feature is also evident at the eastern profile terminus (L9–L10). Consequently, low-velocity sediments attain their greatest depth within the western profile (L1–L3), followed by segments L7–L8 and L9–L10, with the shallowest depths occurring within L4–L6.

The Vs30 distribution along the profile, derived from the inverted velocity structure, reveals that the soil can be categorized into two primary classifications (Figure 12b). The predominant portion of the profile corresponds to category D soil, where Vs30 values range from 200 to 360 m/s. Only a limited segment, comprising the most pronounced high-velocity uplift zones, pertains to category C soil, with Vs30 spanning 360 to 420 m/s.

Collectively, the high-resolution 2D profile (Figure 12a) and Vs30 distribution (Figure 12b) reveal a coherent subsurface architecture, where the 100-m imaging depth is physically validated by the analyzed Rayleigh wavelengths (60–200 m). While deep imaging at segment L5 utilizes spatial interpolation due to low-frequency signal identification limits over shallow bedrock, the overall structural framework remains robust, as the rigid road surface acted as an efficient waveguide for capturing high-frequency traffic-induced vibrations. This multi-scale consistency among DAS results, H/V resonance peaks, and borehole data confirms that the identified alternating uplifts and grabens are genuine geological features, providing a reliable physical foundation for the subsequent discussions on groundwater transport pathways and seismic site effects.

## 4. Discussion

### 4.1. Tectonic and Hydrogeological Implications

The extended velocity profile is in the northern urban sector dominated by the Jinan Pluton. Based on the borehole stratigraphy presented in Figure 11, the high-velocity masses likely correspond to gabbro rock. Consequently, the S-wave velocity profile reveals significant lateral heterogeneity in shallow magma intrusion, exhibiting two prominent igneous bulges and two grabens containing thicker sediment accumulations (Figure 12).

The concealed Qianfoshan Fault and Wenhuaqiao Fault, recognized as nearly parallel structures extending from the southeast to the northwest, intersect the DAS profile. The intersection points spatially coincide, with distinct velocity discontinuities between the two bulges at positions L6 and L9 within the profile (Figure 12), indicating two inclined normal faults. These faults may also exert control over the graben formation. The Lijiaan Fault may pass through from the east side of L4, and a similar wave velocity decrease feature can be seen on the velocity profile as the two faults above. This fault may be a key structural zone that controls the western side of the profile graben.

Although our profile lacks three-dimensional constraints, based on the structural trend of faults running north–south, it can be inferred that there are low-speed structures running north–south within the Pluton. Compared to high-speed rock in the Pluton, groundwater is more likely to flow through them, forming potential groundwater channels.

### 4.2. Seismic Site Effects

Relying solely on Vs30 to classify the soil profile into two categories is insufficient for precise seismic site effect analysis. The shallow structure separates the profile into two bulges and two grabens, each displaying unique H/V patterns. The bulges, from west to east, exhibit H/V peak frequency ranges of 3.5–7 Hz and 2.5–3.5 Hz, respectively, while the grabens show ranges of 1.1–1.7 Hz and 1.7–2.5 Hz individually. By treating the H/V peak frequency as an approximation of soil resonant frequency and estimating the fundamental frequency (f) of reinforced concrete (RC) buildings using f ≈ (46 to 67)/h based on height, the 1–7 Hz range corresponds to RC structures with 2 to 20 stories, which are the most common heights in modern urban environments. Consequently, soil resonant frequencies from 1 to 7 Hz may have potential impacts on mid- to high-rise RC buildings. Therefore, classifying sites into four categories based on velocity structure and H/V distribution allows for a more accurate assessment of the impact of earthquakes on sites, which is beneficial for urban construction planning.

When accounting for the potential impact of hidden geological faults within the urban area, the seismic risk to Jinan City increases substantially. Crucially, the region situated between the Qianfoshan and Wenhuaqiao Faults constitutes the city’s most densely developed and populous district, hosting numerous commercial institutions. A seismic event originating on either fault may generate surface trapped waves within the graben bounded by the two high-velocity uplifted bedrock areas, resulting from the reflection of seismic waves. This phenomenon will amplify and prolong ground vibrations, resulting in significantly amplified destruction across the inter-fault zone.

## 5. Conclusions

Through a comprehensive joint observation experiment that utilized Distributed Acoustic Sensing (DAS) technology and nodal seismometers deployed across Jinan, this study meticulously delineated the spatial variation in shallow sedimentary thickness and identified significant structural undulations, providing critical insights into the local subsurface geology. The results, derived from advanced data integration and analysis, lead to the following key conclusions:

- First, the implementation of mobile DAS using surface-laid fiber-optic cables proves to be a rapid and highly reliable solution for high-resolution urban imaging. By recording relatively short-duration traffic vibrations, we extracted stable dispersion curves and mapped a 100-m S-wave velocity profile that is in good agreement with borehole data. This approach bypasses the coupling issues of pre-existing buried cables and provides a cost-effective, standardized workflow for fine-scale exploration in complex urban environments.
- Second, the survey revealed a distinct “two-uplift, two-graben” tectonic skeleton of the Jinan Pluton, characterized by high-relief topography. The sediment–bedrock interface varies significantly, with uplift peaks buried at only ~20 m and sedimentary thicknesses exceeding 65 m in the graben centers. These structural variations, driven by early magmatic intrusions and subsequent faulting, serve as the primary regulators of the region’s groundwater migration, delineating critical pathways for spring formation and protection.
- Third, the subsurface morphology induces significant seismic site effects, as the profile consists mostly of Class D soils with resonant frequencies spanning 1 Hz to 7 Hz. This frequency range aligns with the fundamental frequencies of 2- to 20-story reinforced concrete buildings, which are prevalent in the study area. Consequently, these findings provide an essential physical basis for urban planning, suggesting that seismic reinforcement and geohazard assessments should prioritize the graben zones to mitigate risks from ground motion amplification and resonance.

In summary, this study demonstrates that the DAS technique, utilizing a rapid surface-laid deployment strategy, provides a transformative and efficient solution for imaging complex urban environments. Even under the suboptimal coupling conditions typical of surface-laid cables, high-precision S-wave velocity structures can be successfully reconstructed from short-duration traffic noise at a significantly lower cost than traditional seismic methods. This mobile DAS strategy constitutes a robust, rapid-exploration framework for urban underground spaces. It not only achieves fine-scale characterization of heterogeneous subsurface structures but also offers a scalable model for urban planning, geohazard mitigation, and the sustainable management of both seismic risks and vital groundwater resources in spring-rich cities globally.

## Data Availability

The raw DAS data and processed results are available from the corresponding author upon reasonable request. These data are not publicly available due to their exceptionally large size (approximately 1 TB), which exceeds the storage capacity of standard public repositories.
